# Epidemiology, Pathogenesis, and Clinical Manifestations of Acute Esophageal Necrosis in Adults

**DOI:** 10.7759/cureus.16618

**Published:** 2021-07-25

**Authors:** Obaid Rehman, Urooj Jaferi, Inderbir Padda, Nimrat Khehra, Harshan Atwal, Mayur Parmar

**Affiliations:** 1 Internal Medicine, Hamilton Medical Center, Dalton, USA; 2 Family Medicine, Hamilton Medical Center, Dalton, USA; 3 Public Health, University of Washington, Seattle, USA; 4 Medicine, Saint James School of Medicine, Arnos Vale, VCT; 5 Basic Sciences, Nova Southeastern University Dr. Kiran C. Patel College Of Osteopathic Medicine, Clearwater, USA

**Keywords:** acute esophageal necrosis (aen), black esophagus, gurvits syndrome, diabetic ketoacidosis, endoscopy, treatment, diagnosis, clinical manifestations, epidemiology, risk factors

## Abstract

Acute esophageal necrosis (AEN), also termed "black esophagus," is a unique and uncommon occurrence observed in severely sick patients. Other terminologies include acute necrotizing esophagitis and Gurvits syndrome. This condition is described as a darkened distal third of the esophagus observed on endoscopy and presents as an upper gastrointestinal (GI) bleed, difficulty swallowing, abdominal pain, fever, syncope, nausea, and vomiting. The etiology of AEN has been linked to several possibilities, such as excessive gastric acid reflux, hypoperfusion, and ischemia due to impaired vascular supply and hemodynamic instability. Risk factors include increased age, sex (male), heart disease, hemodynamic insufficiency, alcohol use, gastric outlet obstruction, diabetic ketoacidosis (DKA), malnutrition, renal disease, and trauma which also have the propensity to complicate disease course. An esophageal biopsy is not warranted. Treatment of AEN is comprised of supportive management with intravenous fluids, proton pump inhibitors (PPI), sucralfate, parenteral nutrition, and antacids. Management of preexisting comorbidities associated with AEN is crucial to prevent exacerbation of the disease course that could result in a poor prognosis and increased mortality rates. This literature review article comprises epidemiology, etiology, pathogenesis, diagnosis, and management of AEN.

## Introduction and background

Acute esophageal necrosis (AEN), also known as the black esophagus, acute necrotizing esophagitis, and Gurvits syndrome, is a rare clinical diagnosis with a relatively poor prognosis [1,2]. Patients are diagnosed with an esophagogastroduodenoscopy (EGD) which shows sections of black-colored esophageal mucosa extending to the distal gastroesophageal junction (GEJ) with possible esophageal extension [1,2]. Acute esophageal necrosis has been linked to several etiologies [3]. It was first associated with upper gastrointestinal bleeding (UGB) in 1990 by Goldenberg et al. and further established as AEN syndrome by Gurvits et al. [3]. Further data from subsequent case reports and case series have allowed a better understanding of this condition. The classic clinical presentation of AEN are patients that present to the emergency room with signs of UGB such as melena, hematemesis, or coffee-ground emesis [3,4]. Acute esophageal necrosis is thought to occur due to one or more of the following causes: ischemic injury to the esophagus, backflow of gastric fluid leading to further injury, and an insufficient mucosal immune response [3,4]. Risk factors include male gender, older age (majority of cases in the sixth decade of life), trauma, and paraesophageal hernia [3,4]. Several comorbidities increase the risk of developing AEN, such as diabetes mellitus (DM), diabetic ketoacidosis (DKA), hypertension, cardiovascular disease, atherosclerotic disease, chronic kidney disease, and chronic liver disease [3,4]. The conventional findings of AEN are associated with the distal 1/3 of the esophagus. This involvement is due to the limited vascular supply corresponding to the upper 2/3 of the esophagus. Histologic involvement of necrosis is not limited to the mucosa but can deeply extend the submucosal and muscularis propria range of the esophagus. In a retrospective study by Day and Sayegh on AEN subjects, it was reported that 32 out of the 310 patients (10.3%) have necrosis extending to the muscularis propria of the distal esophagus. It is vital to note as most studies to date are notable for findings confined to the mucosal and submucosal area [1,2]. A case series reported by Khan et al. followed a 69-year-old male who presented to the emergency department with persistent hematemesis and abdominal pain. An endoscopy was performed, which revealed a black-colored esophagus extending to and suddenly ending at the GEJ junction [5]. The patient was given supportive management with adequate hydration and proton pump inhibitors. The patient displayed moderate recovery of the necrosed esophagus [5]. Fortunately, this patient remained stable and was discharged without any further complications [5]. The current standard first-line management for AEN consists of supportive care with sufficient hydration, proton pump inhibitors, antibiotics, and sucralfate administration subjective to each patient’s needs [6]. Surgical intervention is reserved for patients with a severe presentation with extensive esophageal ulceration, necrosis, and in some instances, esophageal perforation [6]. This article aims to provide an updated review of the epidemiology, etiology, pathogenesis, diagnosis, and management of AEN to better aid clinicians when dealing with this rare condition.

## Review

Pathogenesis and etiology of acute esophageal necrosis

**Figure 1:**
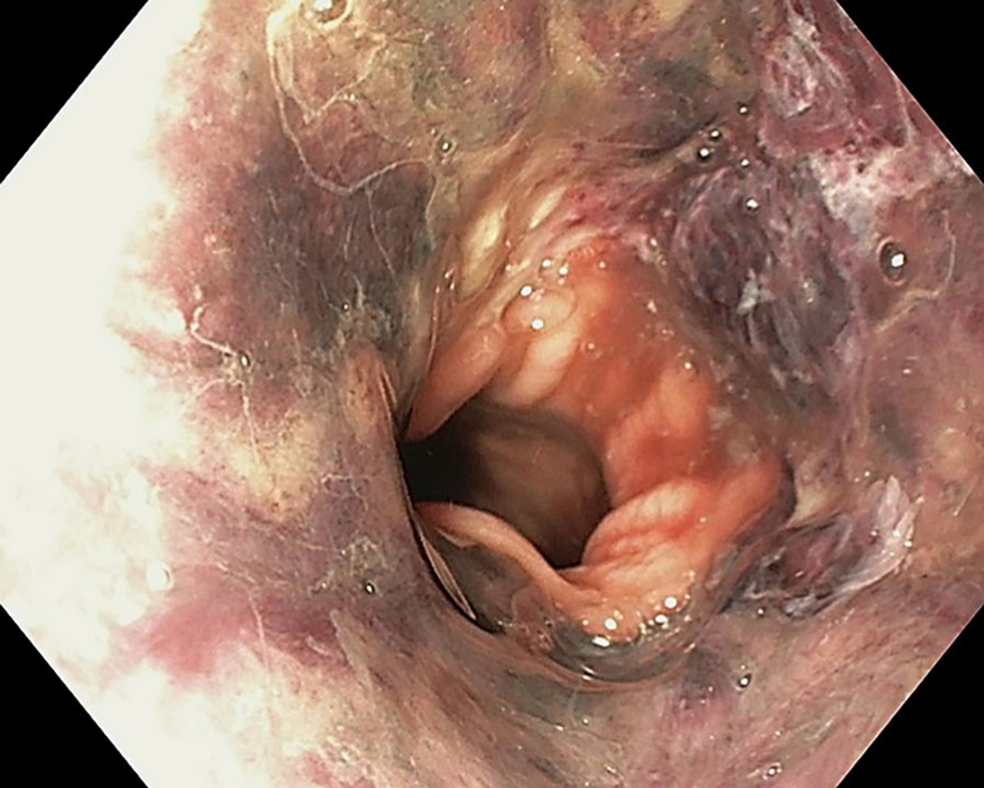
Esophagogastroduodenoscopy showing diffuse and circumferential esophageal necrosis [5] Final, published version of this image is available in the article https://www.karger.com/?doi=10.1159/000496385

The pathogenesis and etiology of AEN are multifactorial that can be related to many factors, such as backflow of gastric contents causing esophageal injury, disruption of the vascular supply leading to hypoperfusion and ischemia, and impaired protective barrier systems due to a weakened immune system and/or a hemodynamically unstable state [6,7]. In a case report study by Khan et al., upper endoscopy revealed AEN (Figure 1), starting at the mid esophagus with an abrupt cutoff at the gastroesophageal junction [5].

Gastroesophageal Reflux Disease

The esophagus is structured with an inner mucosal layer lined by a stratified squamous epithelium, submucosa consisting of connective tissue, layers of muscular tissue, and fibrous tissue followed by an outer connective tissue layer [6,7]. The esophagus is unique from other structures in the gastrointestinal system in that it lacks a serosa layer, which is hypothesized to be protective against necrotic damage that can be inflicted [6,7]. The lining of the stomach mucosa is of columnar epithelium and is distinctly separated in the transition from the esophagus with a zig-zag line [6,7]. Additionally, two muscular sphincters are located in the upper and lower ends of the esophageal wall, with the lower esophageal sphincter (LES) preventing the reflux of stomach acid upward [6,7]. Several factors enhance the insult from backflow of gastric acid, such as a relaxed LES, lower LES pressure, decreased peristaltic movement in the esophagus, and massive influx of gastric contents [8,9]. Medical conditions that enhance these factors are hiatal hernia, obesity, diabetes mellitus, postoperative state, severe obstructive sleep apnea, etc., many of which are also linked with AEN [8,9]. AEN-induced reflux of gastric acid causes an activation of the nociceptors located in the esophageal mucosa that sends a cognitive signal to the central nervous system [8,9]. Naturally, healthy esophageal mucosa consists of a protective barrier for the nociceptors from gastric acid [8,9]. However, with a damaged barrier, the esophageal epithelium is more susceptible to an impaired protective barrier that normally hinders any chemical and necrotic damage [8,9].

Hypoperfusion and Ischemia

The esophagus consists of an abundant blood supply and venous drainage throughout [9,10]. The upper parts of the esophagus, including the upper esophageal sphincter (UES), the parts of the esophagus in the thorax, and lower esophageal parts including the LES, receive blood supply from the inferior thyroid artery, bronchial arteries, and left gastric/phrenic arteries respectively [9,10]. In terms of venous drainage, the upper and middle parts of the esophagus drain into the azygous and hemiazygos veins, and the lower esophagus drains into the left gastric vein [9,10]. The rich blood supply is highly vascularized in the upper and middle portions of the esophagus with sufficient drainage; however, the lower esophagus has more “watershed” zones making it highly susceptible to hypoperfusion and ischemic insult [9,10]. Several risk factors are present in causing low-flow states and ischemic esophageal injury to patients, with the majority of cases of patients consisting of a vascular compromise, such as diabetes mellitus, chronic kidney disease, older age, and hypertension [11]. Other rare causes of hypoperfusion leading to ischemic injury have been reported in a case of ischemic cholangitis leading to the black esophagus, as reported in a case report by Cameron and Schweiger [11]. The case report describes a 62-year-old male patient with a past medical history of coronary artery disease, hypertension, dyslipidemia, type II diabetes mellitus, chronic alcoholism, and hepatitis C presenting with AEN and ischemic cholangitis [11]. The patient was followed up with supportive care and resolved the AEN; however, he developed hepatic failure due to ischemic cholangitis [11]. The authors of this report concluded a poor prognosis due to the combined occurrence of both rare conditions [11].

Weakened Protective Barrier System

Patients with a severely weakened state, such as in cases of malignancy, chronic alcoholism, chronic liver disease and/or cirrhosis, chronic kidney disease, etc., may have a damaged protective barrier system to shield against esophageal injury [11]. Additionally, these patients can have impaired regenerative capabilities of the esophageal epithelium when it is subjected to injury [11].

Epidemiology

Diagnosis of AEN has increased significantly in recent years, possibly due to better implementation and availability of endoscopy [12]. A study comparing 310 consecutive autopsies by Jacobsen et al. displayed evidence of necrosis of 10.3% (32/310) [12]. The patient population in this study was randomly selected, with the age range of the patients being older than two and no upper limit of age [12]. The extension of the necrosis varied amongst the patients with 28.1% (9/32) extending into or through the muscularis propria, 40.6% (13/32) with only mucosal necrosis, and 31.2% (10/32) of patients with necrosis extending into the submucosa [12]. Additionally, the study reported that AEN is usually overlooked and should be considered as a cause for bleeding, perforation, or septicemia [12]. The overall mortality rate of patients presenting with AEN in this study was approximately 32% [12].

Typically, AEN affects males more than females and has a higher predisposition for patients with advanced age [13,14]. In a literature review of all cases of AEN reported by Gurvits et al., out of a total of 88 patients reported with AEN over the last 40 years, 79.5% (70/88) were male, and 18.2% (16/88) were female [13]. The overall mortality rate for all 88 patients was reported as 31.8% [13,14]. A combination of multiple comorbidities and the weakened physical state of a patient can increase the chance of developing AEN significantly [13,14]. The highest occurrence of comorbidities seen in the 88 patients from this literature review was diabetes mellitus with 24% (21/88), malignancy with 20% (18/88), hypertension with 20% (18/88), alcohol abuse with 10% (9/88), and coronary artery disease with 9% (8/88) [13]. 

Differential diagnoses and comorbidities associated with acute esophageal necrosis

**Figure 2:**
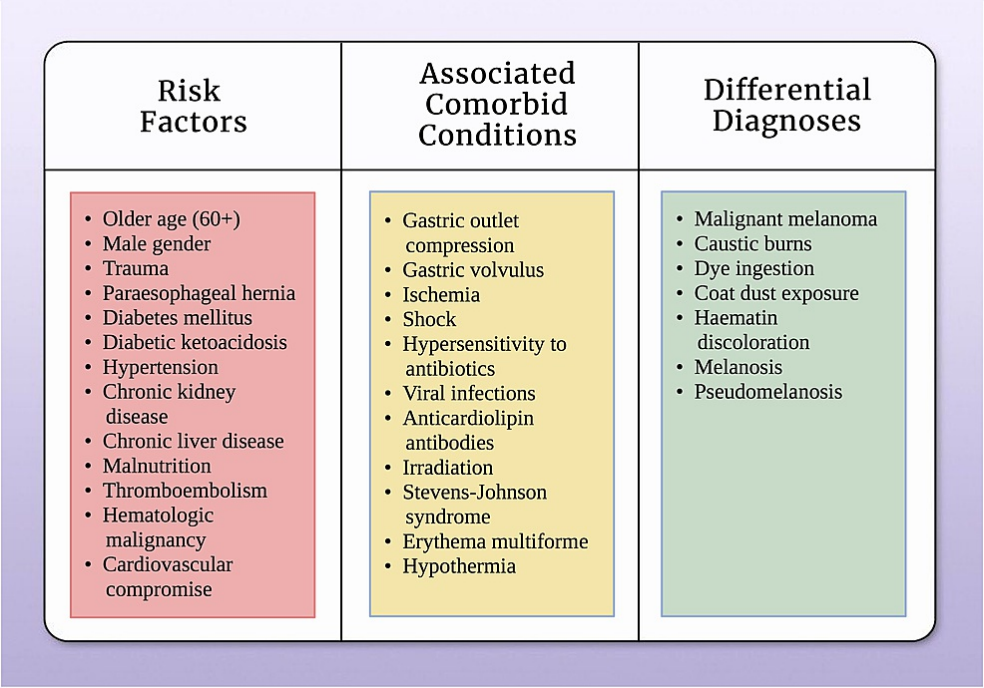
Summary of risk factors, comorbid conditions, and differential diagnoses associated with acute esophageal necrosis

Differentiating AEN from other diagnoses, such as malignant melanoma, acanthosis nigricans, caustic burns, dye ingestion, etc., are crucial in providing appropriate diagnostic procedures and management [3,15]. A thorough depiction of risk factors, associated chronic medical conditions, and differential diagnoses are illustrated in Figure 2.

Collectively, AEN has been associated with many medical conditions, such as gastric outlet obstruction, gastric volvulus, ischemia, shock, hypersensitivity to antibiotics, etc., although a known etiology is not reported [2]. In a retrospective case series, Augusto et al. analyzed the clinical course of 29 patients with AEN and comorbid conditions over 5 years [2]. The age of the patients ranged from 40-91 years of age, with an average age of 75 (16/24). About 83% (24/29) of patients presented with comorbid conditions, and all cases presented with UGB, with approximately 75.9% (22/29) not obtaining hemodynamic stabilization after acute care [2]. However, patients provided with supportive care, proton pump inhibitors, sucralfate therapy (15/29), and broad-spectrum antibiotics (7/29) all had complete resolution of symptoms [2]. Additionally, the researchers reported that advanced age and control of comorbid conditions are more indicative of the prognosis than the extent of esophageal necrosis in all cases [2].

Complications and association with diabetic ketoacidosis

The literature review has shown a possible association between AEN and diabetic ketoacidosis (DKA), as seen in several case reports [16-18]. Acute esophageal necrosis may play a role to the detriment of patients with poorly controlled DKA [16-18]. According to a review by Inayat et al., approximately 90% of patients with AEN were observed to be hyperglycemic when diagnosed with AEN [19]. One of the possible etiologies for this observation is due to hypoperfusion and ischemia that frequently occurs in patients with AEN as the lower third of the esophagus is less vascularized with lower splanchnic blood flow [19]. Patients with diabetes can present with severe hyperglycemia-induced hypovolemia that can exacerbate the ischemia in the lower third of the esophagus [19]. A case study by Thuluvath et al. followed a 30-year-old female patient with a history of type 2 diabetes presenting to the emergency department with symptoms of UGB [20]. On admission, the patient was incoherent, and her status was rapidly deteriorating [20]. The patient was hypotensive (90/50 mmHg), tachycardic (132 bpm), and tachypneic (32/min) with Kussmaul breathing, and complained of abdominal pain on physical examination [20]. Laboratory findings revealed a significant leukocytosis with a white blood cell (WBC) count of 45 K/cu mm (normal: 4.5-11 K/cu mm), hyperglycemia with a blood glucose of 1294 mg/dL (normal: < 140 mg/dL) [20]. Additionally, her findings were consistent with anion gap metabolic acidosis with the bicarbonate level of 4 mmol/L (normal: 23-30 mmol/L), anion gap of 32 mmol/L (normal: <10 mmol/L), and venous blood pH <6.8 (normal: 7.31-7.41) [20]. This patient was diagnosed with DKA and, on EGD, revealed necrosis with black-colored esophageal mucosa at the distal esophagus [20]. This case report, as well as, several others showed a possible association of AEN as a cause of mortality for DKA and can be considered in DKA patients presenting with UGB [20,21].

Clinical management and prognosis

The treatment for AEN is indeterminate [22]. To date, management entails treating the underlying etiology and providing symptomatic relief [22]. Parenteral nutrition (PN) is the preferred route of feeding as opposed to enteral feeding, as irritation to the esophagus may increase the risk of esophageal perforation [22]. Parenteral nutrition ensures adequate nutritional requirements in order to alleviate any irritation to the esophageal mucosal lining and/or the corresponding dysphagia that occurs [22]. A course of 1-2 weeks of PN followed by a transition to a soft diet is the recommended protocol. Adverse effects of PN can include dehydration, electrolyte abnormalities, infection, and thrombosis [22]. Preventative measures such as intravenous fluid administration in esophageal ischemic patients should be considered regardless of the hemodynamic status; this improves vascular perfusion to the less vascularized areas of the lower esophagus and limiting the occurrence of further ischemic tissue damage [22]. Administration of antacids is vital to protect the esophageal mucosal lining from overwhelming gastric acid reflux [22]. Proton pump inhibitors (PPIs) should also be administered intravenously until significant clinical improvement is observed [22]. Once clinical signs and symptoms dramatically improve, therapy can be maintained with oral proton pump inhibitors for a few months [22].

Acute esophageal necrosis cases are subject to biopsy sampling during endoscopy mainly for diagnostic investigation and can be cultured for various pathologies. The presence of pathological microbial cultures in the esophageal region should be treated with either antibacterial, antiviral, or antifungal medications [22]. Broad-spectrum antibiotic management is required for AEN patients who experience fever, exacerbation of disease severity, suspected esophageal perforation, and immunocompromised patients who have preexisting comorbidities associated with AEN as the outcome for these patients is often sepsis with multiorgan failure and death [22]. These preexisting comorbidities include chronic liver disease, chronic kidney disease, and transplant patients [22]. Once the microbial cultures are obtained, management with narrow-spectrum antibiotics can be used to prevent antibiotic-resistant bacteria.

Esophageal bleeding is a cause for concern; thus, submucosal injection of dilute epinephrine can be beneficial [22]. Another method to promptly control active bleeding is by the placement of an endoscopic metallic stent in order to decrease re-bleeding and extent of bleeding [22]. Conversely, balloon tamponade with a Sengstaken-Blakemore tube or nasogastric intubation is contraindicated due to the increased risk of esophageal perforation. In the event of esophageal perforation, surgery is required [22].

Monitoring the esophageal mucosal lining via multiple endoscopies during routine visits is important as there is a likelihood of developing strictures indicative of increased disease severity and progression [22]. In the case of AEN-induced strictures, first-line management such as repeat endoscopic ballooning is standard [22]. In the case of AEN-induced long strictures, endoscopy with adjustable metallic stents has been shown to considerably treat this sequela [22]. If improvements are not notable with repeat endoscopic ballooning, esophageal surgery such as esophagectomy or bypass is the second-line management [22].

Sodium-glucose cotransporter 2 (SGLT2) inhibitors should be cautiously administered in patients with AEN [23]. This drug has a glucose-lowering ability via inhibition of glucose reabsorption in the proximal tubules resulting in increased urinary glucose excretion [23]. SGLT2 inhibitors also initiate natriuresis along with diuresis in patients, which collectively decreases plasma volume, blood pressure, preload, afterload, vascular stiffness, and cardiac-wall stress [23]. A decrease of these factors has beneficial effects on patients with heart failure [23]. However, as SGLT2 inhibitors improve cardiac function by increasing hepatic production of ketones and, thus, worsening DKA, administration of SGLT2 inhibitors is not recommended for DKA [23]. Increased lipolysis and synthesis of ketone bodies can be extremely harmful to type 1 diabetes mellitus patients, including those on low carbohydrate diets [23].

The underlying comorbidities that patients suffering from AEN possess are predominant determinants of the prognosis of AEN [13]. One-third of cases that result in mortality are from underlying medical conditions [13]. In a systemic review, Abdullah et al. reported that over 60% of AEN patients result in a positive outcome with timely management with symptomatic therapy and acid suppression [24]. Important risk factors that may serve as poor prognostic factors include increased age, sex (male), heart disease, hemodynamic insufficiency, alcohol use, gastric outlet obstruction, DKA, malnutrition, renal disease, and trauma [24]. The development of AEN should be considered a poor prognostic factor, correlated with a significant risk of mortality from the underlying comorbidities [24].

Limitations

Our study has a few limitations. Primarily, it is a literature review based on findings reported in peer-reviewed journal articles indexed in PubMed. The peer-reviewed articles on AEN to date are limited based on the rarity of the condition. We have also understood that a differential diagnosis such as caustic burns or esophagitis that may cause bleeding can make a definitive diagnosis of AEN challenging. Literature indicates that AEN cases were found incidentally or on EGF for upper GI bleeding. Acute esophageal necrosis cases may go unnoticed in subjects without bleeding, making the actual prevalence and outcomes difficult. Literature review suggests that there are currently no guidelines for AEN treatment. Emerging research can assist researchers and clinicians in understanding this disease better.

## Conclusions

Acute esophageal necrosis is a rare condition that may occur following recurrent gastric acid reflux, hypoperfusion, and ischemia due to impaired vascular supply, impaired protective barrier systems, and/or hemodynamic instability. The clinical presentation of AEN is a characteristic darkening of the distal-third region of the esophagus, which is observed on endoscopy, hence the term "black esophagus." Management relies heavily on supportive measures, identifying and correcting the hemodynamic compromise, prompt fluid resuscitation alongside PPIs, sucralfate, parenteral nutrition, and antacids. Hemodynamic compromise may result from comorbid conditions such as diabetic ketoacidosis (DKA), malignancy, diabetes mellitus (DM), hypertension, cardiovascular disease, chronic kidney, and liver disease. Monitoring patients for AEN complications such as esophageal perforation is crucial to decrease mortality.

## References

[1] Day A, Sayegh M (2010). Acute oesophageal necrosis: a case report and review of the literature. Int J Surg.

[2] Augusto F, Fernandes V, Cremers MI (2004). Acute necrotizing esophagitis: a large retrospective case series. Endoscopy.

[3] Gurvits GE (2010). Black esophagus: acute esophageal necrosis syndrome. World J Gastroenterol.

[4] Akaishi R, Taniyama Y, Sakurai T, Okamoto H, Sato C, Unno M, Kamei T (2019). Acute esophageal necrosis with esophagus perforation treated by thoracoscopic subtotal esophagectomy and reconstructive surgery on a secondary esophageal stricture: a case report. Surg Case Rep.

[5] Khan H, Ahmed M, Daoud M, Philipose J, Ahmed S, Deeb L (2019). Acute esophageal necrosis: a view in the dark. Case Rep Gastroenterol.

[6] Siddiqi A, Chaudhary FS, Naqvi HA, Saleh N, Farooqi R, Yousaf MN (2020). Black esophagus: a syndrome of acute esophageal necrosis associated with active alcohol drinking. BMJ Open Gastroenterol.

[7] Schizas D, Theochari NA, Mylonas KS (2020). Acute esophageal necrosis: a systematic review and pooled analysis. World J Gastrointest Surg.

[8] Orlando RC (2010). The integrity of the esophageal mucosa. Balance between offensive and defensive mechanisms. Best Pract Res Clin Gastroenterol.

[9] Kim SM, Song KH, Kang SH (2019). Evaluation of prognostic factor and nature of acute esophageal necrosis: restropective multicenter study. Medicine (Baltimore).

[10] Hwang J, Weigel TL (2007). Acute esophageal necrosis: “black esophagus”. JSLS.

[11] Cameron PA, Schweiger F (2017). Forsaken foregut: case report of simultaneous black esophagus and ischemic cholangiopathy. Case Rep Med.

[12] Jacobsen NO, Christiansen J, Kruse A (2003). Incidence of oesophageal necrosis in an autopsy material. APMIS.

[13] Gurvits GE, Shapsis A, Lau N, Gualtieri N, Robilotti JG (2007). Acute esophageal necrosis: a rare syndrome. J Gastroenterol.

[14] Deliwala SS, Bala A, Haykal T, Elbedawi MM, Bachuwa G, Gurvits GE (2020). Acute esophageal necrosis (Gurvits syndrome) presenting as globus and altered phonation. Am J Case Rep.

[15] Shafa S, Sharma N, Keshishian J, Dellon ES (2016). The black esophagus: a rare but deadly disease. ACG Case Rep J.

[16] Field Z, Kropf J, Lytle M, Castaneira G, Madruga M, Carlan SJ (2018). Black esophagus: a rare case of acute esophageal necrosis induced by diabetic ketoacidosis in a young adult female. Case Rep Gastrointest Med.

[17] Worrell SG, Oh DS, Greene CL, DeMeester SR, Hagen JA (2014). Acute esophageal necrosis: a case series and long-term follow-up. Ann Thorac Surg.

[18] Talebi-Bakhshayesh M, Samiee-Rad F, Zohrenia H, Zargar A (2015). Acute esophageal necrosis: a case of black esophagus with DKA. Arch Iran Med.

[19] Inayat F, Hurairah A, Virk HU (2016). Acute esophageal necrosis: an update. N Am J Med Sci.

[20] Thuluvath AJ, Moore D, Alghanim F (2017). Acute esophageal necrosis: a cause or effect of diabetic ketoacidosis (DKA)?. Am J Gastroenterol.

[21] Haghbayan H, Sarker AK, Coomes EA (2018). Black esophagus: acute esophageal necrosis complicating diabetic ketoacidosis. CMAJ.

[22] Dias E, Santos-Antunes J, Macedo G (2019). Diagnosis and management of acute esophageal necrosis. Ann Gastroenterol.

[23] Tomoda Y, Yoshimura D, Hayashida E, Tanaka K (2021). Black Oesophagus-acute esophageal necrosis. QJM.

[24] Abdullah HM, Ullah W, Abdallah M, Khan U, Hurairah A, Atiq M (2019). Clinical presentations, management, and outcomes of acute esophageal necrosis: a systemic review. Expert Rev Gastroenterol Hepatol.

